# Multilayered precision regulation to induce robust CD8^+^ T‐cell immune responses

**DOI:** 10.1002/ctm2.70375

**Published:** 2025-06-18

**Authors:** Yingyue Zeng, Lu Lu, Ji Wang

**Affiliations:** ^1^ School of Life Sciences Liaoning University Shenyang China; ^2^ Key Laboratory of Medical Molecular Virology (MOE/NHC/CAMS), Shanghai Institute of Infectious Disease and Biosecurity, School of Basic Medical Sciences and Shanghai Public Health Clinical Center, Shanghai Frontiers Science Center of Pathogenic Microbes and Infection Fudan University Shanghai China; ^3^ Institute of Precision Medicine, The First Affiliated Hospital Sun Yat‐sen University Guangzhou China

## 1 THE CRITICAL ROLE OF CD8^+^ T CELLS IN CLINICAL APPLICATIONS

Current prophylactic vaccines primarily induce humoral immune responses, which often struggle to effectively combat highly mutable pathogens. For example, if the circulating influenza strain undergoes significant mutation compared with the vaccine strain, vaccine effectiveness can plummet from 60% to as low as 10%. Our study indicated that inducing high levels of tissue‐resident memory CD8^+^ T cells in the respiratory mucosa with a new adjuvant could significantly enhance the ability of existing influenza vaccines to combat variant viruses.^1^

For therapeutic vaccines and tumour immunotherapy, CD8^+^ T cells are even more critical since they are the key effector cells that clear infected or tumour cells. During chronic infection or cancer, the endogenous CD8^+^ T‐cell immune responses may be insufficient in intensity, or dominant epitopes‐specific CD8^+^ T cells may become exhausted and dysfunctional due to prolonged antigen stimulation and other microenvironment cues. Immune checkpoint inhibitor therapy, widely used in clinical practice, can effectively reverse T‐cell exhaustion, but the low response rates (10–30% across tumours) indicate weak endogenous anti‐tumour CD8^+^ T‐cell immune responses in most cancer patients. In such scenarios, inducing CD8^+^ T‐cell immune responses targeting multiple epitopes, especially those subdominant ones, through vaccination might be an effective solution.

## 2 THE SIGNIFICANT GAP BETWEEN CLINICAL NEEDS AND EXISTING TECHNOLOGY

Critically, the threshold of CD8^+^ T cells required for efficacy remains undefined, with no established dose‐response relationship. However, one can look to successful viral vaccines for reference. Antigen‐specific CD8^+^ T‐cell immunity induced by tumour peptide vaccines in clinical studies often accounts for only 0.1% of total CD8^+^ T cells in the blood, which is far below the 10–20% levels achievable by attenuated viral vaccines (e.g., the yellow fever vaccine) and live viral vaccines (e.g., the smallpox vaccine) that can induce lifelong protection.^2^ Judging by these standards, even with very potent adjuvants, existing vaccines still have a considerable gap to bridge in terms of inducing CD8^+^ T‐cell immunity.

## 3 MULTILAYERED PRECISION REGULATION IS REQUIRED

**FIGURE 1:**
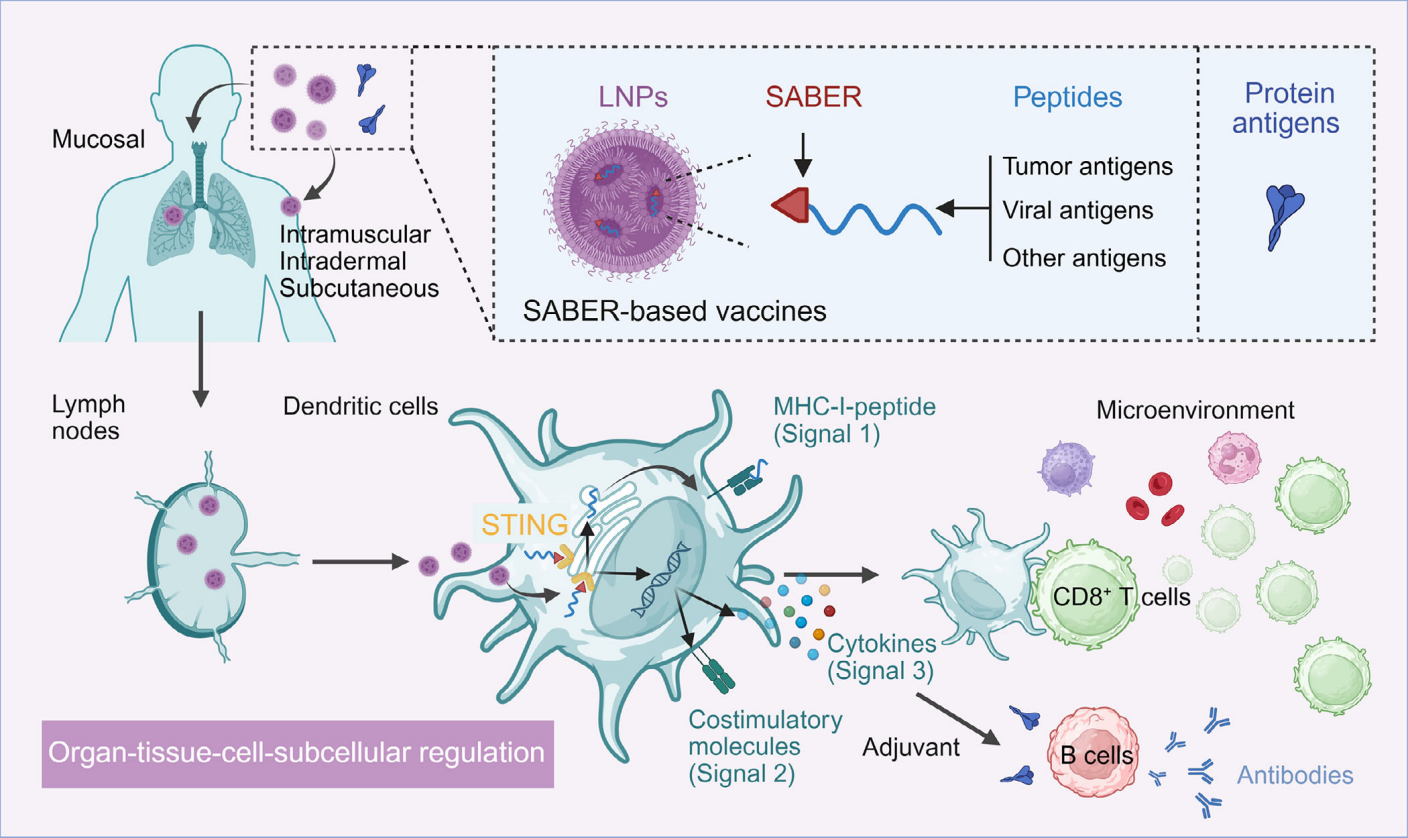
Multilayered precision regulation for CD8^+^ T‐cell immune response. A SABER‐based vaccine consists of SABER‐peptide conjugates encapsulated within LNPs. For infectious disease vaccines, it can also be used in combination with subunit vaccines. The vaccine can be delivered via conventional routes such as intramuscular, intradermal, or subcutaneous injection, or through mucosal administration. Regardless of the delivery method, the vaccine must ultimately be taken up by DCs in peripheral tissues or lymph nodes. Subsequently, the SABER‐peptide complex is transferred into the cytoplasm by LNPs. SABER works in two key ways: first, by binding to STING, it enriches peptides in ER, thereby enhancing antigen cross‐presentation. Second, it activates STING pathways, amplifying the second and third signals. This combined action ultimately leads to the induction of potent CD8^+^ T‐cell immune responses. Furthermore, SABER can also function as an adjuvant, boosting the ability of subunit vaccines to induce antibodies. The schematic diagram was created with BioRender.com. DCs, dendritic cells; LNPs, lipid nanoparticles, SABER, STING agonist‐based ER‐targeting molecules; STING, stimulator of interferon genes.

The difficulty in inducing CD8^+^ T‐cell immune responses stems from the need for precise vaccine delivery and regulation across multiple levels: organ, tissue, cell, and organelles. Only by optimizing each step can ideal results be achieved. At the organ and tissue levels, antigens need to overcome a series of potential physiological barriers, avoid clearance by phagocytes like macrophages, and be efficiently delivered to dendritic cells (DCs), the crucial antigen‐presenting cells for inducing CD8^+^ T‐cell immune responses (Figure 1, signal 1).^1^, ^3^, ^4^ Consequently, a large number of DC‐targeting strategies have been developed over the past 30 years. Lipid nanoparticles (LNPs), widely used in recent mRNA vaccines, have also been found to be effectively engulfed by DCs in lymph nodes.^5^ At the cellular level, antigen presentation is regulated by multiple factors. DCs possess abundant innate immune receptors that can be activated by agonists such as Toll‐like receptors and the stimulator of interferon genes (STING), or respond to nanostructures on microbial or vaccine surfaces, thereby upregulating co‐stimulatory factors (Signal 2) and cytokines (Signal 3) to promote T‐cell proliferation.^6^ Our recent studies have also found that various cells in the microenvironment, such as epithelial cells and erythrocytes, can regulate DC through multiple cytokines and ligands.^1^, ^7^, ^8^

Additionally, precise regulation of antigen presentation at the subcellular level is also crucial. For most low‐affinity epitopes, the process of loading antigen peptides onto major histocompatibility complex class I (MHC‐I) primarily occurs in the endoplasmic reticulum (ER), requiring antigen expression within DCs, or exogenous antigens being transferred into the cytoplasm. Consequently, a vast amount of previous research has focused on improving RNA, DNA, and viral vector technologies to promote efficient antigen expression in DCs, in which we have also done extensive work.^9^, ^10^ Alternatively, new delivery systems have been developed to deliver antigens into the cytoplasm. However, our latest research has found that the efficiency of antigen delivery to ER might be a previously neglected, critical rate‐limiting step in the entire antigen cross‐presentation process. The STING agonist‐based ER‐targeting molecules (SABER) developed in our recent study could greatly enhance the efficiency of this process.^5^

Certainly, a vaccine based on SABER technology is not just a single molecule but a prime example of multilayered precision regulation. A SABER‐based vaccine utilizes LNPs, carriers widely employed in mRNA vaccines, capitalizing on their capability to achieve precise organ‐tissue‐cellular level delivery. Subsequently, the SABER molecule further advances precise regulation to the subcellular level. By harnessing its high affinity for STING, it efficiently enriches antigens in the ER, promoting their “last mile” delivery in cross‐presentation. SABER can simultaneously upregulate co‐stimulatory factors and cytokine expression by activating the STING pathway, substantially enhancing all three signals. Because each step in the immune response induction process is precisely regulated, SABER‐based vaccines possess the ability to induce CD8^+^ T‐cell immune responses comparable to live viral vaccines. In animal models, the CD8^+^ T cells induced against a single epitope reached 30% of total CD8^+^ T cells in the blood, which is 10 times higher than existing potent adjuvants such as Poly‐I:C and ODN1018. This unprecedented capability allows SABER‐based vaccines, even when targeting a single epitope, to demonstrate extraordinary efficacy in challenging tumour models and SARS‐CoV‐2 infection models, laying the foundation for their clinical translation.

Furthermore, it is important to note that beyond its application in CD8^+^ T‐cell vaccines, SABER's STING agonist properties qualify it as an excellent adjuvant. Our research has found that combining SABER‐based vaccines with SARS‐CoV‐2 subunit vaccines can significantly enhance the induction of cross‐protective neutralizing antibodies while inducing high levels of CD8^+^ T‐cell immune responses.

## 4 CLINICAL APPLICATIONS AND CHALLENGES OF MULTILAYERED PRECISION TECHNOLOGY

While multilayered precision regulation can significantly enhance vaccine effectiveness, it inevitably increases the system's complexity. Compared with small molecules or antibodies, the clinical translation of complex systems faces greater challenges. Therefore, we suggest that the design of vaccines based on multilayered precision regulation must consider clinical translation issues from the outset, avoiding the de novo design of new technologies at multiple levels. Instead, technological innovation should be focused on critical points, while mature technologies could be utilized whenever possible. Adhering to these principles, SABER‐based vaccines were designed to employ proven technologies beyond the core innovation. For instance, STING agonists have accumulated extensive data in clinical trials, and LNP technology has been validated in SARS‐CoV‐2 vaccines. Additionally, SABER technology combines ER targeting and immune activation into a single entity, further simplifying the complex system.

While the aforementioned strategies can significantly accelerate the research and translation of next‐generation vaccines and immunotherapies, they require extensive collaboration among academia, hospitals, and industry in terms of technology, intellectual property, and clinical trials — particularly for technologies based on multilayered precision regulation.

## 5 AUTHOR CONTRIBUTIONS

J.W., L.L., and Y.Z. conceptualised and wrote the manuscript.All authors reviewed and approved the final manuscript.

## 6 CONFLICT OF INTEREST STATEMENT

The authors declare no competing interests.
